# Simulating the Conversion of Rural Settlements to Town Land Based on Multi-Agent Systems and Cellular Automata

**DOI:** 10.1371/journal.pone.0079300

**Published:** 2013-11-11

**Authors:** Yaolin Liu, Xuesong Kong, Yanfang Liu, Yiyun Chen

**Affiliations:** 1 School of Resource and Environment Science, Wuhan University, Wuhan, China; 2 Key Laboratory of Geographic Information System, Ministry of Education, Wuhan University, Wuhan, China; Cinvestav-Merida, Mexico

## Abstract

Rapid urbanization in China has triggered the conversion of land from rural to urban use, particularly the conversion of rural settlements to town land. This conversion is the result of the joint effects of the geographic environment and agents involving the government, investors, and farmers. To understand the dynamic interaction dominated by agents and to predict the future landscape of town expansion, a small town land-planning model is proposed based on the integration of multi-agent systems (MAS) and cellular automata (CA). The MAS-CA model links the decision-making behaviors of agents with the neighbor effect of CA. The interaction rules are projected by analyzing the preference conflicts among agents. To better illustrate the effects of the geographic environment, neighborhood, and agent behavior, a comparative analysis between the CA and MAS-CA models in three different towns is presented, revealing interesting patterns in terms of quantity, spatial characteristics, and the coordinating process. The simulation of rural settlements conversion to town land through modeling agent decision and human-environment interaction is very useful for understanding the mechanisms of rural-urban land-use change in developing countries. This process can assist town planners in formulating appropriate development plans.

## Introduction

China is currently experiencing a new stage of urbanization and socio-economic transition [1]. The average growth rate of urbanization is predicted to be approximately 0.8% to 1.0% every year for the next 20 years, and the estimated percentage of urbanization will reach approximately 52% in 2015 and 65% in 2030. The rural-urban relationship is undergoing significant changes because of accelerated urbanization. Additionally, the focus of urbanization in China is shifting from large cities to small towns. The development of small towns reflects the actual process of urbanization in the country. Industrial restructuring and agricultural modernization have resulted in a significant amount of rural labor surplus, thus pushing farmers to convert rural settlements to town land; accordingly, this land conversion has aroused many socio-economic problems [1], [2]. Conversion is an important aspect of the development of a small town, and involves complex decision-making processes that require continuous dynamic geographic analyses to be made by the decision-making agents. Thus, understanding the conversion process of rural settlements to town land is highly important for rural-urban development.

Recently, researchers have paid increasing amount of attention to the construction of models that reveal the mechanism of urban development. Cellular automata (CA), which are spatial-temporal data models, have been widely used to simulate urban expansion [3]. Although CA models can effectively simulate the complex spatial features of urban expansion [4]–[6], the simplicity of the abstract cell and the limitations of these models with respect to incorporating agent behaviors weaken the action of transition rules and restrict further application [3]. Scholars have attempted to integrate CA with Markov processes [7], [8], support vector machines [9], particle swarm optimization [10], ant colony optimization [11], radial basis function neural networks [12], case-based reasoning [13] and multi-agent systems (MAS) [14], [15] to address the aforementioned problems. These CA-based models have demonstrated the effective combination of the geographic environment with social development.

Human are responsible for urban growth, and their behaviors play a significant role in rural-urban land conversion [16]. Human can be classified into different agents according to their social behavior and interests. To achieve a specific purpose, different agents cooperate with each another to make decisions according to their environment. MAS have a prominent advantage in simulating the decision-making process of agents [17]–[19]. The existing MAS-based research has indicated that the quantitative characteristics and spatial structures of urban land can be simulated based on the social, economic, and spatial actions of heterogeneous actors [20]–[24]. A standard protocol for describing agent-based models has been proposed for its continued widespread application [25].

The applications and characteristics of MAS-based models in the simulation of rural-urban land-use changes are summarized in Table 1. Although the literature on MAS applications is extensive, rural settlements are usually neglected or treated as agricultural land in the simulations. The corresponding analysis of farmer behavior is also lacking. A rural settlement is an important land-use type that refers to construction in rural areas in China. The conversion of rural settlements to town land coincides with the lifestyle and identity changes of farmers. Rural settlements are more spatially scattered than urban areas. This scattering results in differences in the spatial characteristics, including transportation costs, land rental values, and living environments. As rational people, stakeholders make decisions to maximize their self-interest with comprehensive consideration given to traffic convenience, environmental conditions, and resource availability in the conversion of rural settlements to town land. Preference conflicts among the stakeholders are inevitable in a dynamic environment. The behavior of farmers is directly associated with the spatial characteristics of the rural settlements. The differences in the spatial characteristics of the scattered rural settlements would increase the probability of preference conflicts and cause difficulty in identifying and solving these conflicts in a simulation. Various interaction rules among the stakeholders will result in different simulation results that increase the uncertainty of the results. An effective simulation model should depict the interaction rules and solve the conflicts with consideration of the geographic environment and the spatial characteristics of the rural settlements. It is necessary to build new interaction rules in MAS by analyzing the specialty of rural settlements. This objective will mark a new attempt for MAS research as well as a complement to its demonstrated applications.

**Table 1:** The main characteristics of rural-urban MAS-based models.

Landscape Types	Agents	Description
Urban land	Household [23], [34]; Regional authority, real estate developer, residents,and environmentalists [17]; Government, residents and propertydevelopers [15].	Agents are divided into different categories based on urban development analysis. Resident behavior is key to building MAS models.
Urban land,agricultural land	Consumers, developers, and farmers [35]; Government, residents,and peasants [36]; Actor agents and facilitators [22], [37].	Research focuses on revealing the socio-economic driving force of land-use changes in the rural-urban fringe. The decision-making behavior of agents is defined in advance.
Agricultural land	Household and landscape agents [38]; Landscape agents andsocial agents [39]; Farmers [40].	Agricultural land conversion is a household-dominated process. Human-landscape systems are built for MAS modeling.

This study aims to build a MAS-CA model for simulating the conversion of rural settlements to town land by projecting new interaction rules among agents. In the model, the driving forces for the spatial patterns of town land expansion from both the perspective of the geographic environment and agent desire are analyzed. Cell state and agent desire are combined to form the interaction rules. Non-construction land and rural settlements are treated differently in the simulation, and the corresponding preference conflicts among agents are coordinated through the continuously iterative process. More detailed information on variables and transition rules are illustrated to develop the proposed model, and the feasibility of the model is verified. To illustrate better the effects of geographic environment, neighborhood, and agent behavior, a comparative analysis between the CA and MAS-CA models is performed in three different towns. The proposed MAS-CA model can offer auxiliary support for the decision-making process of town planners.

## Data and Methods

### Study Area

**Figure 1:**
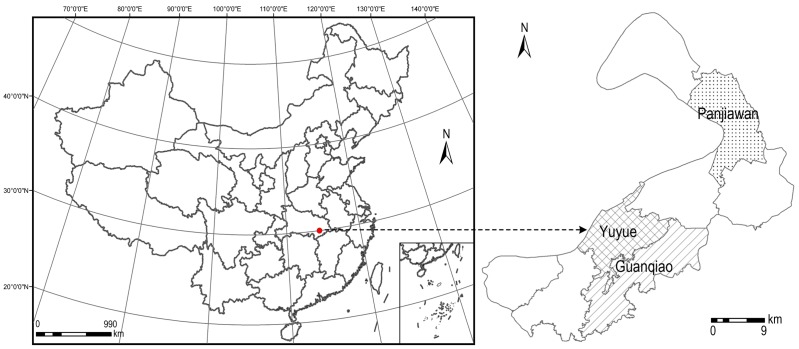
The location of the study area. Yuyue is the central town of Jiayu County. Guanqiao is a hilly industrial town. Panjiawan is an agricultural town located on a plain.

Jiayu County (Figure 1) is located in the southeast of Hubei Province (29°48′∼30°19′S, 113°39′∼114°22′E), and lies on the southern bank of the middle reaches of the Yangtze River. Jiayu County covers an area of 101,842 ha, with 43.70% comprised of plains and 56.30%, hilly areas. The elevation ranges from 19 m to 50 m. Jiayu County consists of eight district towns and has a population of 370,000. As the fastest growing county in Central China, Jiayu County had a growth rate of 12.56% for its gross domestic product between 2000 and 2010. The study area selected for the application of the MAS-CA model includes the towns of Yuyue, Guanqiao, and Panjiawan. Yuyue is the central town of Jiayu County; Guanqiao is a hilly industrial town; and Panjiawan is an agricultural town located on a plain. The study area represents the development of town growth in Central China.

### Data Sources and Processing

The land-use data of the study area include three 1∶10,000 land-use maps (for 2000, 2005 and 2010). Nine land-use types were identified, namely, arable land, gardens, woodland, other agricultural land, town land, rural settlements, other land for construction, water, and abandoned land. The land change constraints were obtained from the general plans for land use of Jiayu County (2005 to 2020). The digital elevation model (DEM) and slope, with a spatial resolution of 30×30 m, were derived from the International Scientific Data Service Platform. The distance variables of CA and the decision-making desires of agents were analyzed using the spatial analyst in ArcGIS 10.0. Before MAS-CA modeling, the land-use data were converted to raster data with a resolution of 30 m×30 m using ArcGIS 10.0. The CA and MAS-CA models were developed using Visual Studio 2010 and Net Framework 3.5, and the spatio-temporal conversion pattern of rural settlements to town land was predicted in three different towns from 2010 to 2020.

### Conceptual Framework and Agent Behavior

**Figure 2:**
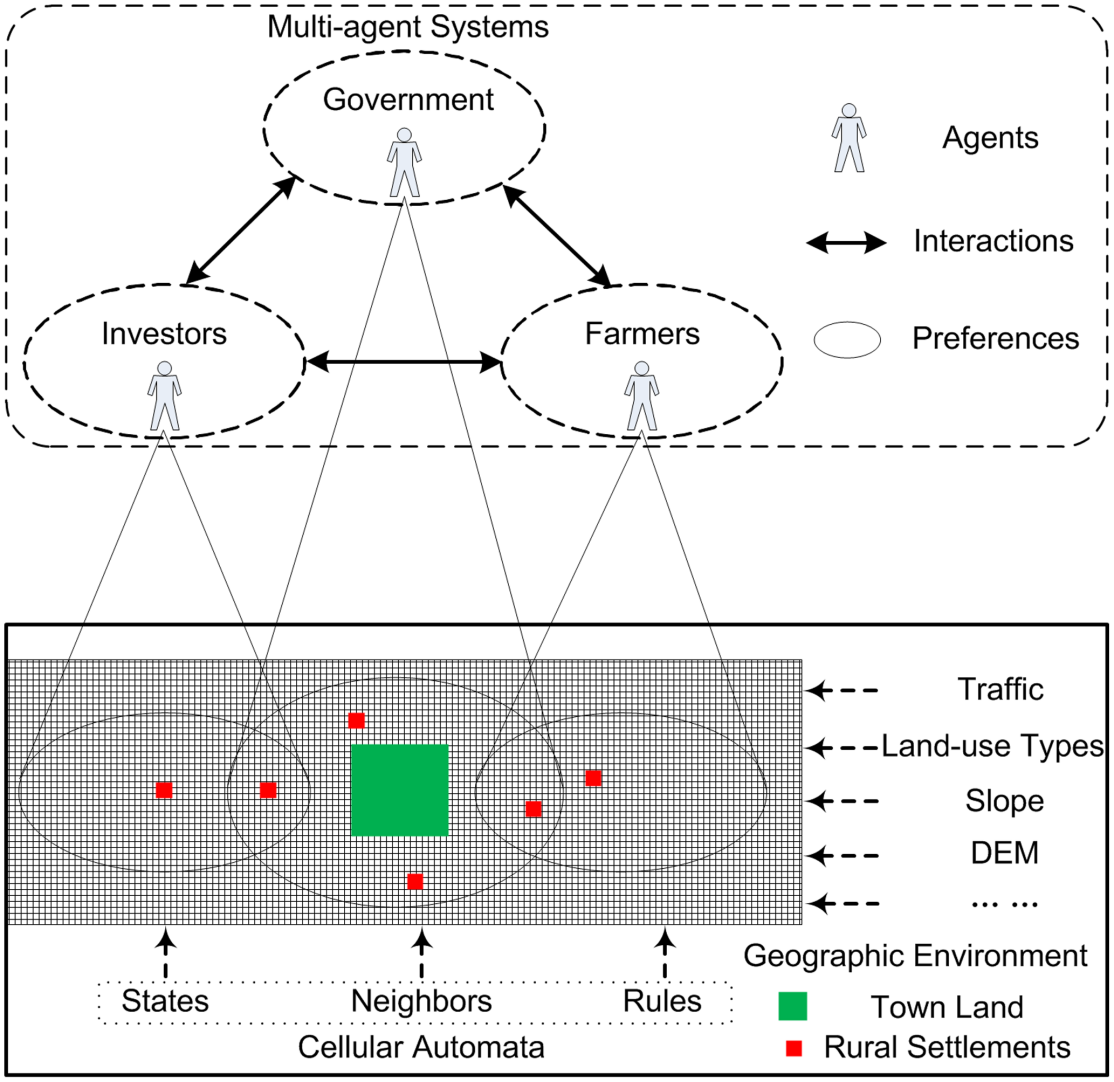
The conceptual framework in the study. The agents, including the government, investors, and farmers, form their self-preference with consideration of the geographic environment and the spatial characteristics of the rural settlements. Combined with the CA transition rules, the interaction rules are projected to solve the preference conflicts among agents.

The MAS-CA model is built based on CA [26] and the Beliefs-Desires-Intentions architecture [17], [22], [27]. Beliefs represent the information acquired from the geographic environment. Desires represent the subjective inclinations of agents. Intentions represent the decision-making behavior of agents. CA is used to simulate town expansion, whereas MAS is used to simulate agent behavior. Both are linked by the interaction rules among agents. The conceptual model includes MAS, CA, and the geographic environment (Figure 2). The geographic environment, including land-use types, distance variables, slope and DEM, is fundamental in modeling, and has been extensively studied [3]. Agents are classified in different groups according to their social attributes. Three different agents are implemented in this study, including the government, investors, and farmers. The conflicts among the agents are more complex because of the differences in the spatial characteristics of the scattered rural settlements. Based on the CA transition rules, the interaction rules are projected to integrate the geographic environment and agent behavior. Therefore, the joint probability of rural settlement conversion to town land is calculated and the spatial layout is simulated.

Guided by experience and knowledge, agents make decisions according to their own characteristics and the surrounding geographic environment. In China, the government plays a leading role in town planning. Permitted built-up areas, conditional built-up areas, and non-built-up areas are planned by the government to guide rational land use. To pursue economic development, the local government often must attract business investment. Consequently, the government provides special consideration to investors for their land requirement. Although farmers have the authority to decide on how to use their rural settlements, their decision-making behaviors are often influenced by the government and investors. Additionally, environmentalists hold a weak position in China. Thus, the decision-making behavior of different agents can vary. Government planning, investor profit and farmer requirement are the most important factors that affect the conversion of rural settlements to town land.

### Process Overview and Scheduling

**Figure 3:**
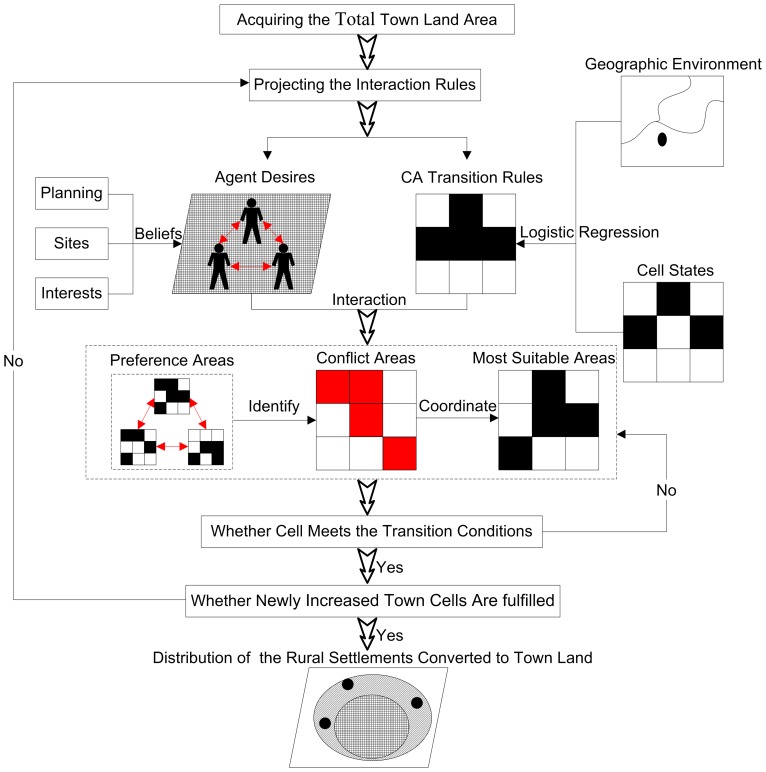
A flowchart illustrating the procedure of MAS-CA model. The total town land areas are acquired in the general plans for land use of Jiayu County (2005 to 2020). Combining the CA transition rules with the desires of agents, the interaction rules are projected to coordinate in the conflict areas. The model iterates according to the interaction rules between agents and their geographic environment. The spatial distribution of the rural settlements converted to town land is formed when the total area of newly increased town land is obtained.

Both CA and MAS are built to highlight the dynamic changes of the complex geographic environment using the “bottom-up” approach. Land-use planning itself is a “top-down” process. The combination of the “bottom-up” and “top-down” process is reflected in agent behavior and CA transition rules. The combination of MAS and CA, which equips each cell with the capacity for social organization and spatial expansion, has better performance for spatial data simulation. Figure 3 displays a flowchart of the simulation process.

#### Step 1: Acquiring the total town land area

The increasing population and rapid development of the social economy have increased the demand for available town land. In this model, the total area of town land and the quota of newly increased town land for each town in 2020 can be acquired in the general plans for land use of Jiayu County (2005 to 2020). The total town land areas are 1,576.68 ha, 170.79 ha and 226.62 ha for Yuyue, Guanqiao and Panjiawan, respectively, and the corresponding newly increased town land areas are 446.49 ha, 43.56 ha and 46.89 ha in the three towns.

#### Step 2: Projecting the interaction rules

By analyzing the desires of agents, cells are assigned with decision attributes, which are the bases for calculating the utility value and selection probability of agents. Combined with the CA transition rules determined by the geographic environment and cell state, the preference areas of each agent for newly increased town land are generated. Different preference conflicts are analyzed and the corresponding coordinated responses are projected. Then the final transition probability of each cell is calculated. The calculated transition probability determines whether a cell is developed into town land. Thus, the transition rules of the MAS-CA model are formed.

#### Step 3: Iteration of the model

The model iterates according to the interaction rules between agents and their geographic environment. The transition probability of each cell is calculated in each iteration. Cells with higher transition probability will be converted into town land. The iteration times are associated with the total areas of newly increased town. The iterations will not end until the total area of the newly increased town land is obtained. The total iteration times (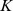) reflect the process of town expansion. The one time iteration in the simulation corresponds to 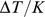 year, where 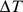 is the observation interval. Lastly, the most suitable areas for conversion into town land are obtained.

#### Step 4: Distribution of the rural settlements converted to town land

The actual 2010 and simulated 2020 town layout maps are overlaid in the Geographic Information System (GIS) environment. Thus, the spatial distribution of rural settlements converted to town land is confirmed for 2010 to 2020.

### Variables and Transition Rules

Two types of variables, cell state and agent desire, are considered in our model. The cell state includes land-use types, slope, DEM, distance variables, and neighbors. Distance variables include the distance to the town center (TownDist), the distance to roads (RoadDist), the distance to water (WaterDist), the distance to the industrial center (IndDist), and the distance to the commercial center (ComDist). These variables are acquired using the spatial analyst in ArcGIS 10.0. Logistic regression is frequently adopted in the acquisition of CA rules and is used to build the suitability maps of spatial variables in the model [3], [26], [28]. Here, logistic regression is used to extract influence coefficients based on town land changes between 2000 and 2005. The logistic regression equation is expressed as follows:
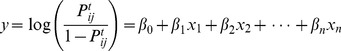
(1)

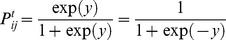
(2)where 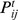 is the transition probability of cell *(i, j)* at the moment *t*; 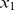, 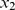, 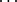 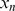 are the driving factors; 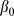 is a constant; 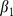, 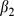, 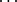 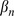 are the corresponding influence coefficients of the driving factors; 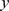 is the logical variable; the undeveloped variable is represented by zero and the developed variable is represented by one. The influence coefficients are calculated in SPSS. Factors that exist the multi-collinearity (variance inflation factor (VIF) >5.0 or tolerance (TOL <0.2) ) or fail the significance test (sig ≥0.05) [26], [29] are removed from the logistic regression model. The regression results of the three towns are presented in Table 2.

**Table 2:** The logistic regression coefficients of the MAS-CA model in the three towns.

	Yuyue			Guanqiao			Panjiawan		
Variable	B	S.E.	Sig	B	S.E.	Sig	B	S.E.	Sig
Constant	2.5645	0.5536	0	2.8774	0.3979	0	5.7523	0.6363	0
TownDist	/	/	/	/	/	/	−0.0055	0.0007	0
RoadDist	−0.0051	0.0006	0	−0.0030	0.0014	0.0327	−0.0033	0.0007	0
WaterDist	−0.0025	0.0004	0	−0.0015	0.0006	0.0101	−0.0010	0.0003	0.0001
IndDist	/	/	/	−0.0022	0.0006	0.0006	/	/	/
ComDist	−0.0004	0.0001	0.0013	/	/	/	/	/	/
DEM	0.0411	0.0190	0.0307	–	–	–	–	–	–
Slope	−0.2128	0.0638	0.0008	–	–	–	0.1622	0.0810	0.0452
PCP	76.14			80.94			79.06		
NR^2^	0.462			0.466			0.443		
-2LL	525.240*			285.903*			440.932*		

Note: PCP = Percentage Correctly Predicted; NR^2^ = Nagelkerke R Square; -2LL = -2 Log Likelihood; B = Beta Coefficient; S.E. = Standard Error; Sig = Significance;/ = VIF >5.0 or TOL <0.2; – = Sig ≥0.05; * = Significant at 0.001.

Agent desire plays an important role in the simulation and can be regarded as the combined decision-making actions among the government, investors, and farmers. A series of agent desires concerning town expansion are analyzed and several representative desires are formulated (Table 3). The proposed agent desires vary with spatial distance. The weights of different desires (Table 4) are acquired from the actual survey of these agents in the three towns (Table S1, Table S2 and Table S3). For example, farmers hope that the newly increased town land observes the proper distance to existing rural settlements. Two reasons are cited for such desire: farmers worry that town expansion will change the lifestyle to which they are accustomed; farmers might benefit from increasing the rental fees in these areas. The agents make their decisions on each cell based on constant information feedback. Each cell is then assigned multiple attributes, which include the geographic environment and agent desires. The cell state is updated after agents make decisions and changes over time according to the predetermined rules [30]. Thereafter, the conversion simulation of rural settlements to town land is performed using a continuously iterative process.

**Table 3:** The agent types and desires for town expansion.

Agents	Desires	
Government	Achieve zone planning (A_1_)	Permitted built-up areas(A_11_)
		Conditional built-up areas (A_12_)
		Non-built-up areas (A_13_)
	Concentrated as much as possible around existing town land (A_2_)	
Investors	Nearby the economic development zone (B_1_)	
	Close to main roads (B_2_)	
	With lower land price (B_3_)	
Farmers	Observe the proper distance to existing rural settlements (C_1_)	0≤ d <200 m (C_11_)
		200 m ≤ d <500 m (C_12_)
		d ≥500 m (C_13_)
	Nearby the existing town land (C_2_)	

**Table 4:** The weights of different desires for expansion of the three towns.

	Yuyue	Guanqiao	Panjiawan
A_1_	0.917	0.888	0.796
A_11_	0.779	0.767	0.713
A_12_	0.221	0.233	0.288
A_13_	0	0	0
A_2_	0.083	0.113	0.204
B_1_	0.583	0.456	0.358
B_2_	0.283	0.313	0.296
B_3_	0.133	0.231	0.346
C_1_	0.720	0.774	0.745
C_11_	0.205	0.151	0.177
C_12_	0.595	0.657	0.666
C_13_	0.200	0.191	0.157
C_2_	0.280	0.226	0.255

According to the desires and weights presented in Table 3 and Table 4, the preference probability of each agent for cell *(i, j)* can be described by the following formula:
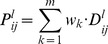
(3)where 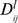 is the individual desire observed from the no. *l* agent group (*l* can be one, two, or three in this case) for cell *(i, j)*; *k* is the serial number of desires; *m* is the total number of desires for the no. *l* agent group; and 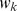
is the weight coefficient of the number *k* desire.

The transition rules are designed at two levels, the CA transition rule and the agent decision-making rule. At the level of the CA transition rule, the development probability of cell *(i, j)* at moment *t +1* is determined by the cell state. The land space is divided into regular cells and is conceptualized using the Moore neighborhood in this model, which is comprised of 3×3 square cells. The transition probability of the kernel cell is influenced by the state of its neighbors. More neighbors in the state of the town land correspond to a higher transition probability for the cell. The neighbor function is expressed as the following:
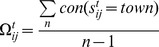
(4)where 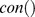 is a conditional function. If the state of 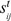 is the town land at moment *t*, the value returns to one; otherwise, the value is equal to zero. Based on the overall consideration of the driving factors, neighbor function, and random factors in town development, the formula for the CA model is constructed as the following [9], [11]:
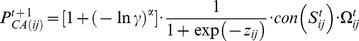
(5)where 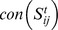 is the natural constraint condition that can be either zero or one. In this study, we hypothesized that town land cannot be converted into other land-use types; water and basic farmland cannot be converted into town land; 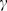 is a random value between zero and one; and 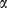 controls the random value [31].

However, the CA transition rule is insufficient for exploring the conversion process of rural settlements to town land. Agent desire should be considered appropriately. Desires differ among different agents in each cell; thus, preference conflicts are inevitable among agents. Solving these conflicts is the core of the transition rule design. Based on the CA transition rule, government, investors, and farmers create preference areas for their own interests in each iteration. Then, the preference conflicts and the corresponding cells are identified. Two cases are considered, namely the unanimous preference areas and inconsistent preference areas. There are no significant conflicts for unanimous preference areas, whereas the conflicts must be coordinated for inconsistent preference areas. Preference conflicts are classified into eight types (Table 5). The preference probability of agent *l* is influenced by the preferences of the other agents. The agents adjust their preference probability according to the conflict types.

**Table 5:** The preference conflicts among different agents in the conversion of rural settlements to town land.

Conflict Types	Government	Investors	Farmers
*Both vs. Farmers (a)*	0	0	1
*Government vs. Both (b)*	0	1	1
*Investors vs. Both (c)*	1	0	1
*Both vs. Government (d)*	1	0	0
*Both vs. Investors (e)*	0	1	0
*Farmers vs. Both (f)*	1	1	0
*Unanimous Agreement (g)*	1	1	1
*Unanimous Disagreement (h)*	0	0	0

Note: 1 = Agree; 0 = Disagree.

The final transition probability of cell *(i, j)* can be represented as the following equation:
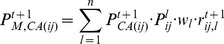
(6)where 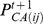 is the CA transition probability of cell *(i, j)* at moment *t +1*; 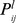 is the preference probability of agent *l* for cell *(i, j)*; 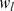 is the decision weight coefficient of agent *l*; and 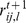 is the coordinating coefficient of agent *l* for cell *(i, j)* at moment *t +1*, which can be expressed as follows:
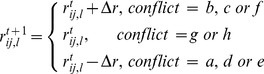
(7)where 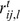 is the coordinating coefficient of agent *l* for cell (*i*, *j*) at moment *t*, with an initial value of one; 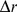 is the interaction value; and *a* to *h* are the conflict types.

Two cases are considered in the model, namely, rural settlements and non-construction land, to illustrate better the preference conflicts in the conversion of rural settlements to town land. The transition probabilities of both cases are calculated according to Eq. (6). Co-deciding using different decision powers is a common type of planning [22]. These two cases differ in their decision weights. The final transition probability is directly associated with the decision weights. With the power to allocate and dispose town land, the government in China has more decision power than investors or farmers. Therefore, the decision weight of the government is higher than the others for both cases, and the decision power of farmers should be stressed for rural settlements. Based on the Analytic Hierarchy Process (AHP) and the Delphi method (Table S4, Table S5 and Table S6), the decision weights of each agent group are acquired for the simulation (Table 6).

**Table 6:** The decision weights of agents for the MAS-CA simulation in the three towns.

	Non-construction Land			Rural Settlements		
	Government	Investors	Farmers	Government	Investors	Farmers
Yuyue	0.544	0.297	0.159	0.499	0.252	0.249
Guanqiao	0.487	0.309	0.204	0.463	0.289	0.247
Panjiawan	0.466	0.293	0.241	0.459	0.286	0.254

## Results

### Model Evaluation

**Figure 4:**
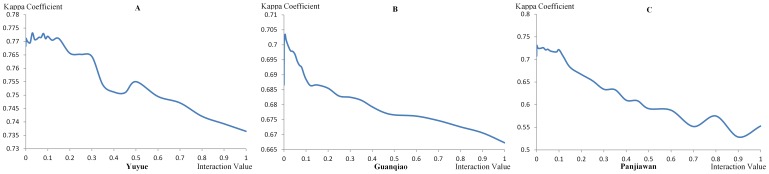
The relationship between interaction value and the Kappa coefficient of MAS-CA in the three towns. The interaction values, ranging from 0 to 1, are used for the validation of the relationship between interaction value and the Kappa coefficient in Yuyue (A), Guanqiao (B), and Panjiawan (C), respectively.

In this study, the CA and MAS-CA models are both implemented to compare the differences among the simulations. The solely CA model runs according to Eq. (5). Both models are parameterized based on the land-use data of 2000 and 2005. We simulated the town expansion from 2005 to 2010 in the three towns and compared the actual 2010 results with the simulated 2010 results to validate the accuracy of the simulation. The simulation accuracy is examined using the Kappa coefficient [17], [32]. The interaction value of the MAS-CA model is tested to acquire a reasonable value through multiple experiments. As shown in Figure 4, the interaction value between 0 and 0.1 holds a higher Kappa value. The interaction values with the maximum Kappa value are 0.03, 0.005 and 0.001 in Yuyue, Guanqiao, and Panjiawan, respectively.

**Figure 5:**
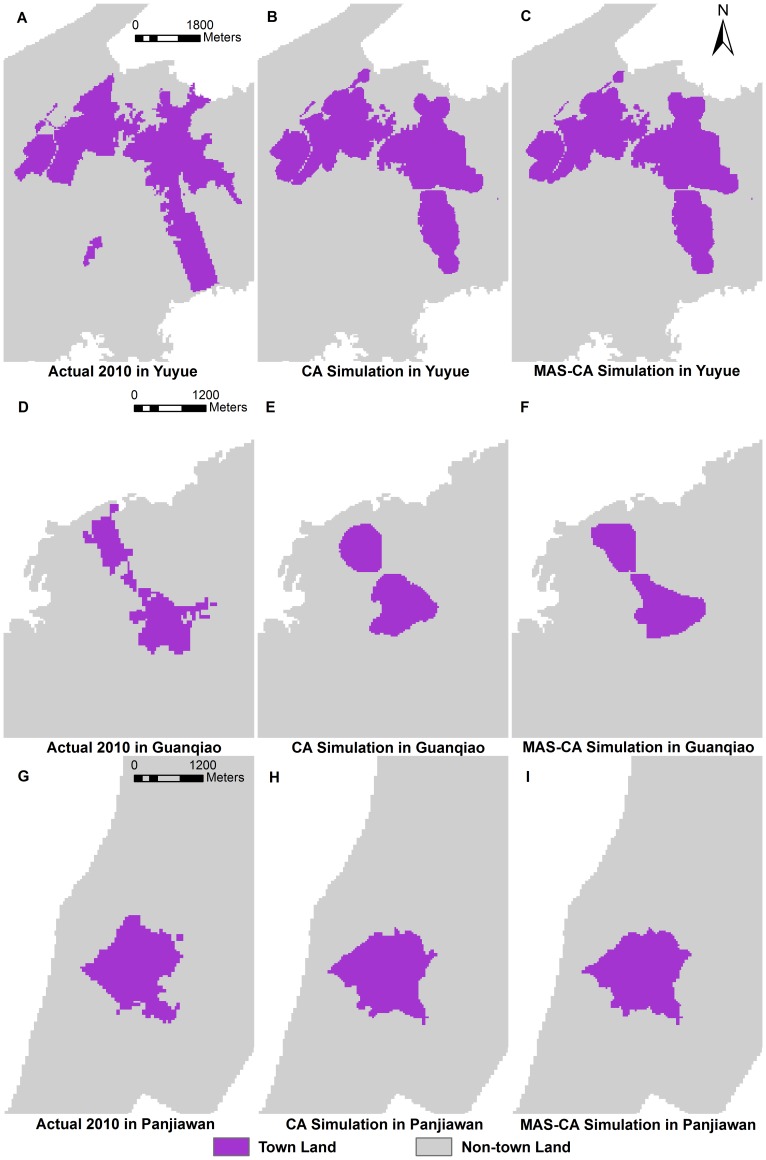
The actual town land and simulated town land for the three towns in 2010, based on CA and MAS-CA. (A) Actual town land for Yuyue in 2010; (B) Simulated town land for Yuyue using CA in 2010; (C) Simulated town land for Yuyue using MAS-CA in 2010; (D) Actual town land for Guanqiao; (E) Simulated town land for Guanqiao using CA in 2010; (F) Simulated town land for Guanqiao using MAS-CA in 2010; (G) Actual town land for Panjiawan in 2010; (H) Simulated town land for Panjiawan using CA in 2010; (I) Simulated town land for Panjiawan using MAS-CA in 2010.

We calculated the Kappa coefficient based on the simulated 2010 and actual 2010 results in the three towns (Table 7). To illustrate more effectively the validation of the MAS-CA model, we ran 60 model realizations in each town to analyze the variation of the Kappa coefficient (Table S7). The stochastic disturbance term has a subtle influence on the simulation results, which can help to generate a more realistic pattern in the MAS-CA simulation. Therefore, the simulation results come from one realization in this study. The Kappa coefficients indicate that the MAS-CA model is feasible and authentic for simulating town expansion. Compared with the CA model, the MAS-CA model presents a significant improvement, especially for Guanqiao. The spatial pattern of the MAS-CA simulation (Figure 5F) is more consistent with the actual 2010 results (Figure 5D) than the CA simulation (Figure 5E) in Guanqiao, whereas the spatial differences between the MAS-CA simulation (Figures 5C and 5I) and the CA simulation (Figures 5B and 5H) are relatively minor in Yuyue and Panjiawan. Guanqiao is a hilly industrial town, where the interactions of agents have more influence on town expansion. The Kappa coefficients for the MAS-CA simulation in Yuyue, Guanqiao, and Panjiawan are 0.7732, 0.7034 and 0.7312, respectively. These values are higher than those obtained from the CA simulation. The overall results of the MAS-CA simulation are acceptable.

**Table 7:** The Kappa coefficients of CA and MAS-CA in the three towns.

Models	Yuyue	Guanqiao	Panjiawan
CA	0.7580	0.5523	0.6851
MAS-CA	0.7732	0.7034	0.7312

### Scenario Simulations

**Figure 6:**
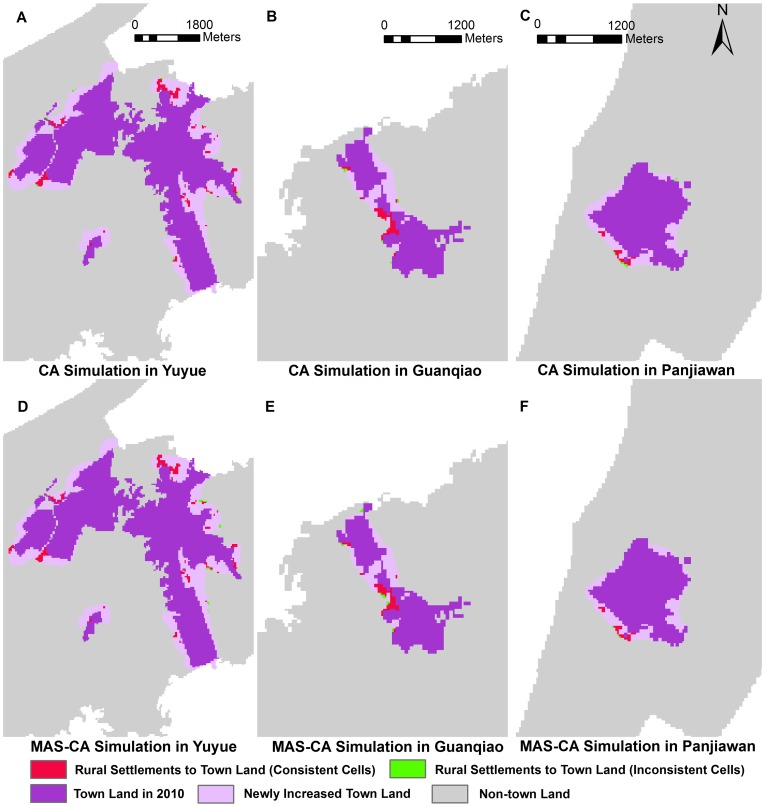
Scenario simulations for the conversion of rural settlements to town land for the three towns using CA and MAS-CA from 2010 to 2020. Rural settlements converted to town land are presented with green cells and red cells. The green cells indicate the inconsistent conversion of rural settlements to town land between CA and MAS-CA simulation, whereas the red cells indicate the consistent cells. (A) CA simulation in Yuyue; (B) CA simulation in Guanqiao; (C) CA simulation in Panjiawan; (D) MAS-CA simulation in Yuyue; (E) MAS-CA simulation in Guanqiao; (F) MAS-CA simulation in Panjiawan.

According to the transition rules, the expansion of the three towns from 2010 to 2020 is simulated based on the CA and MAS-CA models. We overlaid the simulated 2020 results with the actual 2010 results to acquire the simulated 2020 results for the conversion of rural settlements to town land using the spatial overlay analysis in GIS (Figure 6).

As shown in Figure 6, the CA simulation is similar to the MAS-CA simulation in shape. However, the characteristics of rural settlement conversion to town land vary in the three towns. The total area of rural settlement converted to town land in Yuyue from the CA simulation is 39.15 ha, whereas the area is 40.05 ha in the MAS-CA simulation. Fifty-eight cells (5.22 ha) differ in locations (the blue cells in Figures 6A and 6D). Differences in the quantity and location also exist in Guanqiao (Figures 6B and 6E) and Panjiwan (Figures 6C and 6F). The inconsistent cells are mainly distributed in the fringe of the newly built-up areas. This result indicates that preference conflicts among the agents occur frequently in these areas.

The newly increased town land in Yuyue, with an area of 446.49 ha, is far larger than that in Guanqiao (43.56 ha) and Panjiawan (46.89 ha), resulting in the conversion of more rural settlements into town land. The Jiayu Economic Development Zone, which is located in southeastern Yuyue, provides unique geographic superiority and numerous benefits to investors. Farmers prefer to transform their rural settlements into town land for higher profits [33]. Thus, the Jiayu Economic Development Zone appears as a “hot spot” in the conversion of rural settlements to town land. For Guanqiao and Panjiawan, the lower values of newly increased town land result in the comparatively less conversion of rural settlements into town land. Unlike the town expansion in Panjiawan, the newly increased town land is mainly clustered in the northwestern region of the existing town land in Guanqiao, where the enterprises are concentrated. The influence of geographic environment is considered in the transition rules and affects both the CA and MAS-CA models. Given the topographic differences, the shape of the newly increased town land in Panjiawan is more regular than that in Guanqiao.

For the MAS-CA model, the simulation of town expansion is a dynamic coordinating process that is reflected in model iteration. According to the transition rules, the model iterates to achieve the total areas of newly increased town land from 2010 to 2020. The iteration times are 300, 230 and 250 for Yuyue, Guanqiao and Panjiawan, respectively (Figure S1). The one time iteration in the simulation is 1/30 year for Yuyue, 1/23 year for Guanqiao and 1/25 year for Panjiawan. Different preference conflicts have different transition characteristics in the three towns (Table 8). It can be observed that the conflict area of rural settlements is less than that of non-construction land. Specifically, the conflict area are 11.25 ha, 9.54 ha and 4.23 ha for the combined conflicts *b*, *d*, *e* and *f* in Yuyue, Guanqiao and Panjiawan, respectively, and the corresponding transition areas are 4.23 ha, 5.22 ha and 1.98 ha. In comparison with non-construction land, conflicts *a* and *c* for rural settlements are missing in the process of town expansion. The most common conflicts for rural settlements that occur in the three towns are *e* and *f*. This phenomenon is in accordance with actual planning in China. The government plans the permitted built-up areas, which usually include the preference areas of the investors. Farmers are more susceptible in these two conflicts. This implies the transformation of *e* to *f* and then to *g*. Additionally, it should be noted that the simulation involves the process of identifying the cells with unanimous preference of the agents (conflicts *g* and *h*).

**Table 8:** Quantitative characteristics for conversion of R & NL to town land in different conflicts (ha).

	Yuyue				Guanqiao				Panjiawan			
	R		NL		R		NL		R		NL	
	CA	TA	CA	TA	CA	TA	CA	TA	CA	TA	CA	TA
*a*	0	0	33.39	5.04	0	0	11.61	0.18	0	0	8.28	0.36
*b*	0.54	0.36	30.15	25.56	0.18	0.09	11.52	7.56	0.63	0.54	13.41	9.09
*c*	0	0	16.65	12.51	0	0	1.80	1.71	0	0	7.20	4.95
*d*	4.50	0.63	18.27	2.97	0.90	0.18	5.13	0.90	2.07	0.36	4.95	0.81
*e*	3.06	0.18	33.75	6.66	6.30	2.79	9.63	4.32	0.63	0.18	13.23	4.77
*f*	3.15	3.06	24.12	23.85	2.16	2.16	5.22	5.22	0.90	0.90	7.56	7.56
*g*	35.82	35.82	329.85	329.85	4.05	4.05	14.31	14.31	1.71	1.71	15.66	15.66
*h*	37.08	0	272.97	0	28.80	0	281.43	0	12.96	0	193.41	0
Tot	84.15	40.05	759.15	406.44	42.39	9.27	340.65	34.20	18.90	3.69	263.70	43.20

Note: R = Rural Settlements; NL = Non-construction Land; CA = Conflict Area; TA = Transition Area.

**Figure 7:**
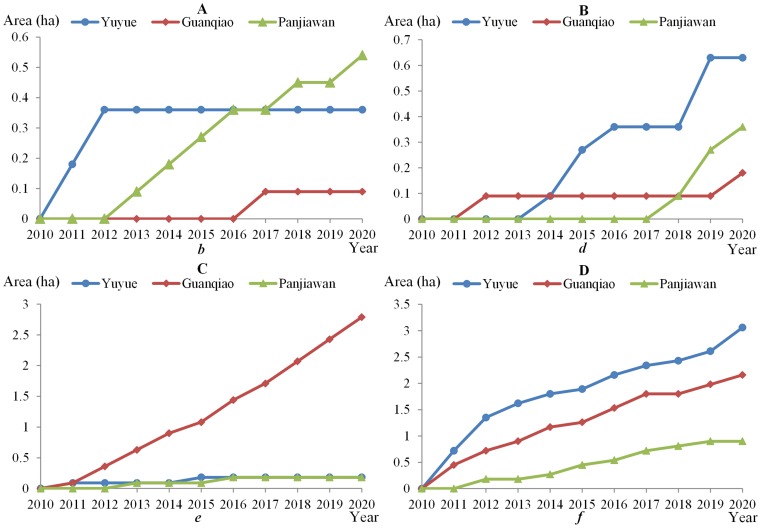
The coordinating process of conflicts *b*, *d*, *e* and *f* for the conversion of rural settlements to town land in the three towns from 2010 to 2020. The areas of rural settlements converted to town land in the three towns are calculated for conflicts *b* (*Government vs. Both*), *d* (*Both vs. Government*), *e* (*Both vs. Investors*) and *f* (*Farmers vs. Both*). The area is enlarged over time. (A) The coordinating process of conflicts *b*; (B) The coordinating process of conflicts *d*; (C) The coordinating process of conflicts *e*; (D) The coordinating process of conflicts *f*.

The coordinating process of the conversion of rural settlements to town land for conflicts *b*, *d*, *e* and *f* is shown in Figure 7. Conflict *f* presents the most continuous coordinating process, and the coordinating area accounts for 72.34%, 41.38%, and 45.45% of the total coordinating area of rural settlements for the combined conflicts *b*, *d*, *e* and *f* in Yuyue, Guanqiao and Panjiawan, respectively. There are apparent differences in the coordinating process for conflicts *b*, *d* and *e* in the three towns. Conflict *b* is well resolved in Panjiawan, and similarly for conflict *d* in Yuyue and conflict *e* in Guanqiao. These conflict characteristics are associated with the function and development orientation of towns. The guidance power of governmental planning is insufficient for influencing investors and farmers in agricultural towns. When conflict *b* occurs, the government tends to compromise with investors and farmers in Panjiwan. In contrast, as the central town of Jiayu County, Yuyue has a clear plan in guiding town development. Investors and farmers are more easily affected by the government in conflict *d*. The economic development is largely determined by investor demand in industrial towns. This results in the high coordination for conflict *e* in Guanqiao. Different conflicts are well coordinated through the agent interaction in the three towns.

**Figure 8:**
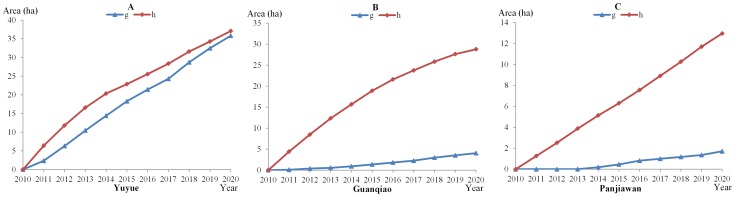
The iteration process of conflicts *g* and *h* for the conversion of rural settlements to town land in the three towns. The areas of rural settlements in conflicts *g* (*unanimous agreement*) and *h* (*unanimous disagreement*) are calculated in Yuyue (A), Guanqiao (B), and Panjiawan (C). The area is enlarged over time.

The area of the rural settlements in conflicts *g* and *h* increases continuously in the iteration process (Figure 8). In Yuyue, the largest increase in town land results in the largest area of rural settlements in conflicts *g* and *h*. It can be observed that the increase in rural settlements varies significantly between conflicts *g* and *h*. The area of the rural settlements in conflict *h* is larger than that in conflict *g*, especially for Guanqiao and Panjiawan. The results indicate that different agents can more easily reach an agreement to veto the conversion rather than accept it. In reality, the dynamic interaction among agents is well reflected in the MAS-CA simulation.

## Discussion

In this study, we present a coupled MAS-CA model to for simulating the spatial pattern of the conversion of rural settlements to town land. The combination of MAS and CA equips each fixed cell with the capabilities of social organization and spatial expansion. The MAS-CA modeling process consists of two contents, namely, conceptual construction and computational design. The conceptual construction determines whether the real world is expressed reasonably, whereas the computational design determines the accuracy of the simulation. The proposed MAS-CA model differs from the general CA model in its transition rule. Interactions between the geographic environment and agents are combined with neighbor effects in the model.

Despite the complexity of rural-urban land conversion, the proposed MAS-CA model demonstrates the relationship between the geographic environment and agents. The preference conflicts among agents are clearly classified into eight types. A coordinating coefficient is introduced into the model to form a new transition rule in the modeling process, making the interaction among agents close to reality. Agents communicate and influence each other in each cell to determine whether the cell should be converted into town land. This marks a new attempt in the integration of CA with MAS to simulate the conversion of rural settlements to town land. The quantity, spatial characteristics, and coordination process of the conversion of rural settlements to town land are thoroughly presented in the present study. The results indicate that the preference conflicts demonstrate different coordinating characteristics in different towns. Town planners can formulate appropriate development plans according to the preference conflicts of the specific agents in different towns.

Although the proposed MAS-CA model is effective for simulations, the model possesses several limitations for practical applications. First, the MAS-CA model only considers cases in which other land-use types are converted to town land and ignores the conversion of town land to other land-use types because of socio-economic development and environmental protection reasons. Inevitably, this fact results in the reduced accuracy of the simulation. Second, we only consider some of the major desires and preference conflicts among agents. However, the conflicts and interaction among agents are very complex and changeable in reality. Investors can be subdivided into estate investors and other enterprise investors to reflect the differences in their desires. The universal agent desires for all three towns may decrease the difference between towns. The interaction rule among agents is a simplification with respect to reality in the model. Third, the simulated results fail to present the newly increased town land, which grows as an independent development. Thus, the leapfrogging development of town land cannot be properly reflected. This phenomenon can be observed in Yuyue (Figures 5A, 5B and 5C). The newly increased town land is related to the neighbor diffusion of CA rules as well as the uncertain development of policy. These limitations should be elaborated and solved in future studies using the MAS-CA model.

In summary, the proposed MAS-CA model is a powerful tool for the simulation of the conversion of rural settlement to town land, incorporated the influences of agents and the geographic environment. This model aids in our understanding of the mechanism of rural-urban land conversion. Further research should focus on the study of individual behavior modes among different agents at a micro-scale level. The conversion between town land and rural settlements could be better understood by assessing the simulation results.

## Supporting Information

Figure S1 The iteration simulation of town land expansion in the three towns from 2010 to 2020. The iteration simulation for Yuyue is presented in Figures A to E; The iteration simulation for Guanqiao is presented in Figures F to J; The iteration simulation for Panjiawan is presented in Figures K to O; T is the iteration time.
(TIF)

Table S1 Investigation on the weights of government desires in the three towns.
(DOC)

Table S2 Investigation on the weights of investor desires in the three towns.
(DOC)

Table S3 Investigation on the weights of farmer desires in the three towns.
(DOC)

Table S4 The decision weights of agents calculated by AHP.
(DOC)

Table S5 Reference interval values for Delphi method based on AHP.
(DOC)

Table S6 The decision weights of agents calculated by Delphi method.
(DOC)

Table S7 The variation of Kappa coefficient for 60 model realizations in the three towns.
(DOC)
