# Supplementary figures and images for: Simulating the Conversion of Rural Settlements to Town Land Based on Multi-Agent Systems and Cellular Automata
Source: PLoS One. 2013 Nov 11;8(11):e79300. doi: 10.1371/journal.pone.0079300 (PMC3823707; doi:10.1371/journal.pone.0079300)

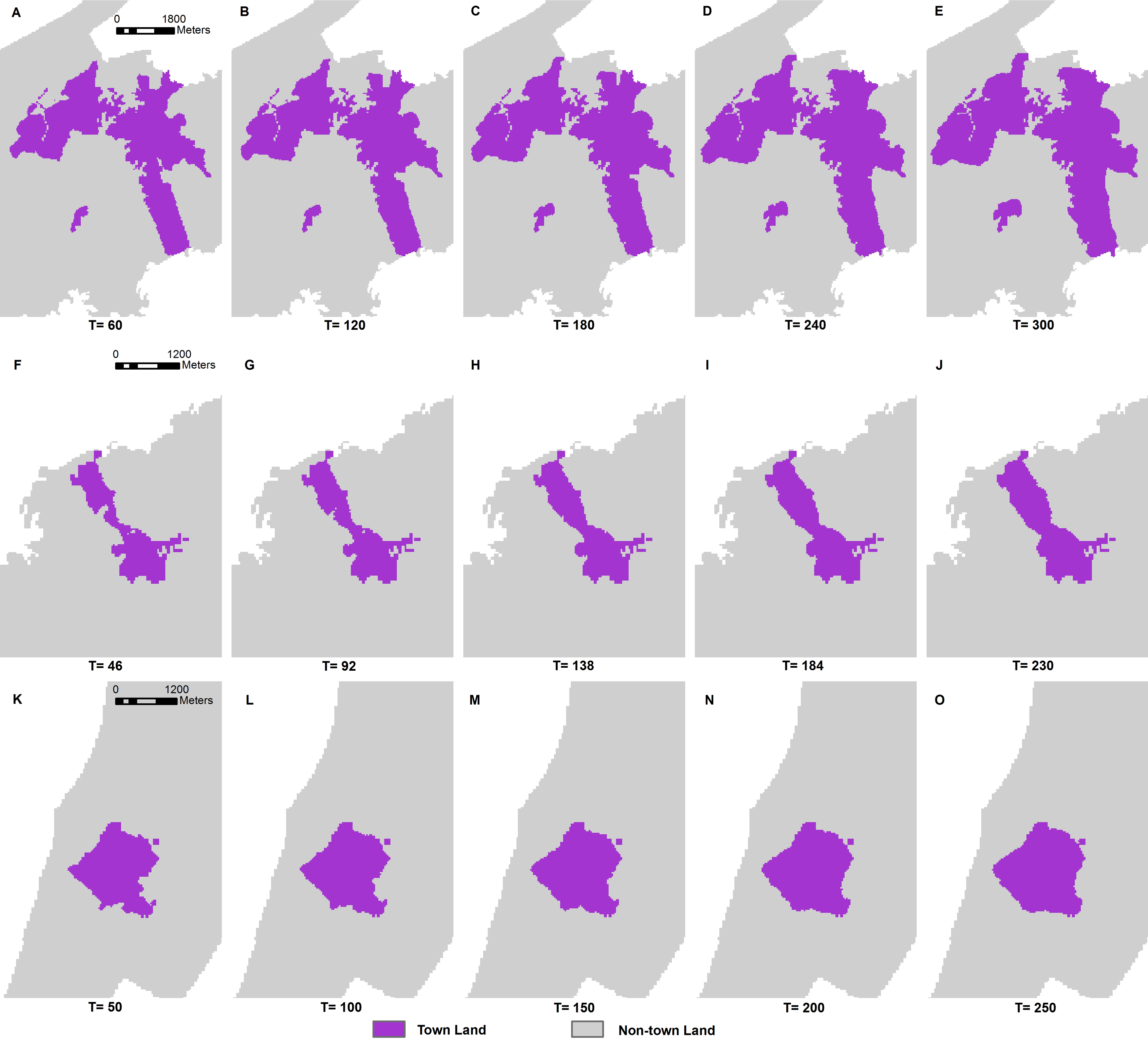

Supplement: Figure S1 — The iteration simulation of town land expansion in the three towns from 2010 to 2020. The iteration simulation for Yuyue is presented in Figures A to E; The iteration simulation for Guanqiao is presented in Figures F to J; The iteration simulation for Panjiawan is presented in Figures K to O; T is the iteration time. (TIF) [file pone.0079300.s001.tif]
