# Supplementary material for: Simulating the Conversion of Rural Settlements to Town Land Based on Multi-Agent Systems and Cellular Automata
Source: PLoS One. 2013 Nov 11;8(11):e79300. doi: 10.1371/journal.pone.0079300 (PMC3823707; doi:10.1371/journal.pone.0079300)
Supplement: Table S1 — Investigation on the weights of government desires in the three towns. (DOC) [file pone.0079300.s002.doc]

| **Table S1. Investigation on the weights of government desires in the three towns.** | | | | | | | | | | | | | | | |
| --- | --- | --- | --- | --- | --- | --- | --- | --- | --- | --- | --- | --- | --- | --- | --- |
|  | | | | | | | | | | | | | | | |
|  | **Yuyue** | | | | | **Guanqiao** | | | | | **Panjiawan** | | | | |
| Number | A_1_ | A_2_ | A_11_ | A_12_ | A_13_ | A_1_ | A_2_ | A_11_ | A_12_ | A_13_ | A_1_ | A_2_ | A_11_ | A_12_ | A_13_ |
| 1 | 0.90 | 0.10 | 0.80 | 0.20 | 0 | 0.90 | 0.10 | 0.75 | 0.25 | 0 | 0.75 | 0.25 | 0.65 | 0.35 | 0 |
| 2 | 0.95 | 0.05 | 0.80 | 0.20 | 0 | 0.85 | 0.15 | 0.80 | 0.20 | 0 | 0.85 | 0.15 | 0.60 | 0.40 | 0 |
| 3 | 1.00 | 0 | 0.90 | 0.10 | 0 | 0.85 | 0.15 | 0.85 | 0.15 | 0 | 0.85 | 0.15 | 0.55 | 0.45 | 0 |
| 4 | 0.85 | 0.15 | 0.85 | 0.15 | 0 | 0.90 | 0.10 | 0.75 | 0.25 | 0 | 0.85 | 0.15 | 0.70 | 0.30 | 0 |
| 5 | 0.90 | 0.10 | 0.75 | 0.25 | 0 | 0.85 | 0.15 | 0.60 | 0.40 | 0 | 0.80 | 0.20 | 0.75 | 0.25 | 0 |
| 6 | 0.90 | 0.10 | 0.80 | 0.20 | 0 | 1.00 | 0 | 0.85 | 0.15 | 0 | 0.75 | 0.25 | 0.80 | 0.20 | 0 |
| 7 | 0.90 | 0.05 | 0.90 | 0.10 | 0 | 0.95 | 0.05 | 0.75 | 0.25 | 0 | 0.80 | 0.20 | 0.70 | 0.30 | 0 |
| 8 | 0.90 | 0.10 | 0.70 | 0.30 | 0 | 0.85 | 0.15 | 0.75 | 0.25 | 0 | 0.75 | 0.25 | 0.75 | 0.25 | 0 |
| 9 | 0.90 | 0.10 | 0.75 | 0.25 | 0 | 0.85 | 0.15 | 0.80 | 0.20 | 0 | 0.80 | 0.20 | 0.70 | 0.30 | 0 |
| 10 | 1.00 | 0 | 0.80 | 0.20 | 0 | 0.90 | 0.10 | 0.85 | 0.15 | 0 | 0.80 | 0.20 | 0.80 | 0.20 | 0 |
| 11 | 0.90 | 0.10 | 0.65 | 0.35 | 0 | 0.90 | 0.10 | 0.70 | 0.30 | 0 | 0.75 | 0.25 | 0.80 | 0.20 | 0 |
| 12 | 0.85 | 0.15 | 0.65 | 0.35 | 0 | 0.85 | 0.15 | 0.75 | 0.25 | 0 | 0.80 | 0.20 | 0.75 | 0.25 | 0 |
| Mean | 0.917 | 0.083 | 0.779 | 0.221 | 0 | 0.888 | 0.113 | 0.767 | 0.233 | 0 | 0.796 | 0.204 | 0.713 | 0.288 | 0 |
