# Supplementary material for: Simulating the Conversion of Rural Settlements to Town Land Based on Multi-Agent Systems and Cellular Automata
Source: PLoS One. 2013 Nov 11;8(11):e79300. doi: 10.1371/journal.pone.0079300 (PMC3823707; doi:10.1371/journal.pone.0079300)
Supplement: Table S2 — Investigation on the weights of investor desires in the three towns. (DOC) [file pone.0079300.s003.doc]

| **Table S2. Investigation on the weights of investor desires in the three towns.** | | | | | | | | | | |
| --- | --- | --- | --- | --- | --- | --- | --- | --- | --- | --- |
|  | | | | | | | | | | |
|  | **Yuyue** | | | | **Guanqiao** | | | **Panjiawan** | | |
| Number | B_1_ | B_2_ | B_3_ | B_1_ | | B_2_ | B_3_ | B_1_ | B_2_ | B_3_ |
| 1 | 0.55 | 0.35 | 0.10 | 0.50 | | 0.30 | 0.20 | 0.35 | 0.35 | 0.30 |
| 2 | 0.50 | 0.30 | 0.20 | 0.40 | | 0.35 | 0.25 | 0.30 | 0.25 | 0.45 |
| 3 | 0.45 | 0.25 | 0.30 | 0.35 | | 0.25 | 0.40 | 0.35 | 0.30 | 0.35 |
| 4 | 0.55 | 0.35 | 0.10 | 0.65 | | 0.30 | 0.05 | 0.40 | 0.20 | 0.40 |
| 5 | 0.60 | 0.25 | 0.15 | 0.45 | | 0.50 | 0.05 | 0.35 | 0.20 | 0.45 |
| 6 | 0.50 | 0.45 | 0.05 | 0.55 | | 0.20 | 0.25 | 0.35 | 0.25 | 0.40 |
| 7 | 0.65 | 0.20 | 0.15 | 0.35 | | 0.35 | 0.30 | 0.45 | 0.40 | 0.15 |
| 8 | 0.50 | 0.45 | 0.05 | 0.25 | | 0.40 | 0.35 | 0.30 | 0.25 | 0.45 |
| 9 | 0.60 | 0.35 | 0.05 | 0.45 | | 0.30 | 0.25 | 0.45 | 0.35 | 0.20 |
| 10 | 0.55 | 0.35 | 0.10 | 0.65 | | 0.25 | 0.10 | 0.30 | 0.35 | 0.35 |
| 11 | 0.55 | 0.30 | 0.15 | 0.45 | | 0.40 | 0.15 | 0.35 | 0.35 | 0.30 |
| 12 | 0.65 | 0.25 | 0.10 | 0.35 | | 0.45 | 0.20 | 0.35 | 0.30 | 0.35 |
| 13 | 0.40 | 0.35 | 0.25 | 0.45 | | 0.25 | 0.30 |  |  |  |
| 14 | 0.80 | 0.10 | 0.10 | 0.55 | | 0.20 | 0.25 |  |  |  |
| 15 | 0.65 | 0.15 | 0.20 | 0.45 | | 0.25 | 0.30 |  |  |  |
| 16 | 0.75 | 0.20 | 0.05 | 0.45 | | 0.25 | 0.30 |  |  |  |
| 17 | 0.70 | 0.15 | 0.15 |  | |  |  |  |  |  |
| 18 | 0.55 | 0.30 | 0.15 |  | |  |  |  |  |  |
| Mean | 0.583 | 0.283 | 0.133 | 0.456 | | 0.313 | 0.231 | 0.358 | 0.296 | 0.346 |
