# Supplementary material for: Simulating the Conversion of Rural Settlements to Town Land Based on Multi-Agent Systems and Cellular Automata
Source: PLoS One. 2013 Nov 11;8(11):e79300. doi: 10.1371/journal.pone.0079300 (PMC3823707; doi:10.1371/journal.pone.0079300)
Supplement: Table S3 — Investigation on the weights of farmer desires in the three towns. (DOC) [file pone.0079300.s004.doc]

| **Table S3. Investigation on the weights of farmer desires in the three towns.** | | | | | | | | | | | | | | | |
| --- | --- | --- | --- | --- | --- | --- | --- | --- | --- | --- | --- | --- | --- | --- | --- |
|  | | | | | | | | | | | | | | | |
|  | **Yuyue** | | | | | **Guanqiao** | | | | | **Panjiawan** | | | | |
| Number | C_1_ | C_2_ | C_11_ | C_12_ | C_13_ | C_1_ | C_2_ | C_11_ | C_12_ | C_13_ | C_1_ | C_2_ | C_11_ | C_12_ | C_13_ |
| 1 | 0.80 | 0.20 | 0.20 | 0.70 | 0.10 | 0.75 | 0.25 | 0.15 | 0.55 | 0.30 | 0.85 | 0.15 | 0.15 | 0.65 | 0.20 |
| 2 | 0.75 | 0.25 | 0.25 | 0.50 | 0.25 | 0.85 | 0.15 | 0.10 | 0.80 | 0.10 | 0.65 | 0.35 | 0.10 | 0.70 | 0.20 |
| 3 | 0.70 | 0.30 | 0.20 | 0.60 | 0.20 | 0.80 | 0.20 | 0.15 | 0.75 | 0.10 | 0.75 | 0.25 | 0.20 | 0.55 | 0.25 |
| 4 | 0.90 | 0.10 | 0.30 | 0.70 | 0 | 0.85 | 0.15 | 0 | 0.90 | 0.10 | 0.65 | 0.35 | 0.15 | 0.65 | 0.20 |
| 5 | 0.50 | 0.50 | 0.10 | 0.80 | 0.10 | 0.75 | 0.25 | 0 | 0.85 | 0.15 | 0.85 | 0.15 | 0.20 | 0.60 | 0.20 |
| 6 | 0.55 | 0.45 | 0.20 | 0.70 | 0.10 | 0.65 | 0.35 | 0.15 | 0.65 | 0.20 | 0.60 | 0.40 | 0.15 | 0.75 | 0.10 |
| 7 | 0.65 | 0.35 | 0.20 | 0.60 | 0.20 | 0.75 | 0.25 | 0.15 | 0.65 | 0.20 | 0.80 | 0.20 | 0.15 | 0.65 | 0.20 |
| 8 | 0.85 | 0.15 | 0.15 | 0.65 | 0.20 | 0.65 | 0.35 | 0.20 | 0.55 | 0.25 | 0.70 | 0.30 | 0.25 | 0.55 | 0.20 |
| 9 | 0.75 | 0.25 | 0.25 | 0.55 | 0.20 | 0.70 | 0.30 | 0.30 | 0.35 | 0.35 | 0.50 | 0.50 | 0.35 | 0.60 | 0.05 |
| 10 | 0.65 | 0.35 | 0.30 | 0.40 | 0.30 | 0.80 | 0.20 | 0.20 | 0.55 | 0.25 | 0.80 | 0.20 | 0.20 | 0.65 | 0.15 |
| 11 | 0.60 | 0.40 | 0.30 | 0.55 | 0.15 | 0.85 | 0.15 | 0.25 | 0.50 | 0.25 | 0.75 | 0.25 | 0.25 | 0.60 | 0.15 |
| 12 | 0.75 | 0.25 | 0.20 | 0.65 | 0.15 | 0.90 | 0.10 | 0.20 | 0.50 | 0.30 | 0.85 | 0.15 | 0.30 | 0.65 | 0.05 |
| 13 | 0.55 | 0.45 | 0.10 | 0.80 | 0.10 | 0.75 | 0.25 | 0.10 | 0.65 | 0.25 | 0.90 | 0.10 | 0.15 | 0.75 | 0.10 |
| 14 | 0.75 | 0.25 | 0.15 | 0.75 | 0.10 | 0.85 | 0.15 | 0.10 | 0.75 | 0.15 | 0.65 | 0.35 | 0.25 | 0.65 | 0.10 |
| 15 | 0.85 | 0.15 | 0.20 | 0.60 | 0.20 | 0.70 | 0.30 | 0.10 | 0.75 | 0.15 | 0.85 | 0.15 | 0.15 | 0.65 | 0.20 |
| 16 | 0.75 | 0.25 | 0.20 | 0.75 | 0.05 | 0.85 | 0.15 | 0.15 | 0.65 | 0.20 | 0.75 | 0.25 | 0.15 | 0.70 | 0.15 |
| 17 | 0.50 | 0.50 | 0.30 | 0.40 | 0.30 | 0.75 | 0.25 | 0.10 | 0.80 | 0.10 | 0.75 | 0.25 | 0.20 | 0.60 | 0.20 |
| 18 | 0.90 | 0.10 | 0.35 | 0.35 | 0.30 | 0.80 | 0.20 | 0.15 | 0.65 | 0.20 | 0.65 | 0.35 | 0.25 | 0.50 | 0.25 |
| 19 | 0.85 | 0.15 | 0.20 | 0.70 | 0.10 | 0.85 | 0.15 | 0.15 | 0.75 | 0.10 | 0.60 | 0.40 | 0.20 | 0.65 | 0.15 |
| 20 | 0.80 | 0.20 | 0.10 | 0.70 | 0.20 | 0.75 | 0.25 | 0.10 | 0.65 | 0.25 | 0.90 | 0.10 | 0.10 | 0.80 | 0.10 |
| 21 | 0.55 | 0.45 | 0.10 | 0.80 | 0.10 | 0.65 | 0.35 | 0.15 | 0.55 | 0.30 | 0.85 | 0.15 | 0.15 | 0.65 | 0.20 |
| 22 | 0.80 | 0.20 | 0.15 | 0.70 | 0.15 | 0.75 | 0.25 | 0.20 | 0.65 | 0.15 | 0.75 | 0.25 | 0.15 | 0.70 | 0.15 |
| **Table S3. Continued** | | | | | | | | | | | | | | | |
|  | | | | | | | | | | | | | | | |
|  | **Yuyue** | | | | | **Guanqiao** | | | | | **Panjiawan** | | | | |
| Number | C_1_ | C_2_ | C_11_ | C_12_ | C_13_ | C_1_ | C_2_ | C_11_ | C_12_ | C_13_ | C_1_ | C_2_ | C_11_ | C_12_ | C_13_ |
| 23 | 0.70 | 0.30 | 0.25 | 0.50 | 0.25 | 0.85 | 0.15 | 0.05 | 0.85 | 0.10 | 0.65 | 0.35 | 0.20 | 0.70 | 0.10 |
| 24 | 0.75 | 0.25 | 0.20 | 0.60 | 0.20 | 0.90 | 0.10 | 0 | 0.90 | 0.10 | 0.85 | 0.15 | 0.15 | 0.65 | 0.20 |
| 25 | 0.70 | 0.30 | 0.25 | 0.50 | 0.25 | 0.85 | 0.15 | 0.25 | 0.50 | 0.25 | 0.75 | 0.25 | 0.30 | 0.50 | 0.20 |
| 26 | 0.75 | 0.25 | 0.30 | 0.40 | 0.30 | 0.65 | 0.35 | 0.10 | 0.75 | 0.15 | 0.85 | 0.15 | 0.10 | 0.80 | 0.10 |
| 27 | 0.90 | 0.10 | 0.25 | 0.45 | 0.30 | 0.75 | 0.25 | 0.20 | 0.65 | 0.15 | 0.75 | 0.25 | 0.05 | 0.90 | 0.05 |
| 28 | 0.65 | 0.35 | 0.20 | 0.50 | 0.30 | 0.85 | 0.15 | 0.05 | 0.85 | 0.10 | 0.55 | 0.45 | 0.10 | 0.65 | 0.25 |
| 29 | 0.85 | 0.15 | 0.10 | 0.60 | 0.30 | 0.75 | 0.25 | 0.15 | 0.70 | 0.15 | 0.90 | 0.10 | 0.15 | 0.65 | 0.20 |
| 30 | 0.55 | 0.45 | 0.10 | 0.30 | 0.60 | 0.55 | 0.45 | 0.25 | 0.50 | 0.25 | 0.80 | 0.20 | 0.20 | 0.70 | 0.10 |
| 31 | 0.65 | 0.35 | 0.15 | 0.65 | 0.20 | 0.85 | 0.15 | 0.25 | 0.55 | 0.20 | 0.75 | 0.25 | 0.15 | 0.65 | 0.20 |
| 32 | 0.85 | 0.15 | 0.20 | 0.60 | 0.20 | 0.85 | 0.15 | 0.25 | 0.50 | 0.25 | 0.85 | 0.15 | 0.15 | 0.70 | 0.15 |
| 33 | 0.65 | 0.35 | 0.25 | 0.60 | 0.15 | 0.75 | 0.25 | 0.15 | 0.55 | 0.30 | 0.80 | 0.20 | 0.20 | 0.65 | 0.15 |
| 34 |  |  |  |  |  | 0.65 | 0.35 | 0.20 | 0.65 | 0.15 | 0.75 | 0.25 | 0.10 | 0.80 | 0.10 |
| 35 |  |  |  |  |  | 0.85 | 0.15 | 0.25 | 0.60 | 0.15 | 0.65 | 0.35 | 0.15 | 0.70 | 0.15 |
| 36 |  |  |  |  |  |  |  |  |  |  | 0.70 | 0.30 | 0.15 | 0.70 | 0.15 |
| 37 |  |  |  |  |  |  |  |  |  |  | 0.55 | 0.45 | 0.20 | 0.65 | 0.15 |
| Mean | 0.720 | 0.280 | 0.205 | 0.595 | 0.200 | 0.774 | 0.226 | 0.151 | 0.657 | 0.191 | 0.745 | 0.255 | 0.177 | 0.666 | 0.157 |
