# Supplementary material for: Simulating the Conversion of Rural Settlements to Town Land Based on Multi-Agent Systems and Cellular Automata
Source: PLoS One. 2013 Nov 11;8(11):e79300. doi: 10.1371/journal.pone.0079300 (PMC3823707; doi:10.1371/journal.pone.0079300)
Supplement: Table S4 — The decision weights of agents calculated by AHP. (DOC) [file pone.0079300.s005.doc]

| **Table S4. The decision weights of agents calculated by AHP.** | | | | |
| --- | --- | --- | --- | --- |
|  |  |  |  |  |
|  | Government | Investors | Farmers | *W_l_* |
| Government | 1 | 2 | 3 | 0.540 |
| Investors | 1/2 | 1 | 2 | 0.297 |
| Farmers | 1/3 | 1/2 | 1 | 0.163 |
| Note: λ_max_= 3; Consistency Index = 0; Random Index = 0.58; Consistency Ratio < 0.1. | | | | |
