# Supplementary material for: Simulating the Conversion of Rural Settlements to Town Land Based on Multi-Agent Systems and Cellular Automata
Source: PLoS One. 2013 Nov 11;8(11):e79300. doi: 10.1371/journal.pone.0079300 (PMC3823707; doi:10.1371/journal.pone.0079300)
Supplement: Table S5 — Reference interval values for Delphi method based on AHP. (DOC) [file pone.0079300.s006.doc]

| **Table S5. Reference interval values for Delphi method based on AHP.** | | | |
| --- | --- | --- | --- |
|  | | | |
|  | Government | Investors | Farmers |
| Interval Values | 0.440~0.640 | 0.197~0.397 | 0.063~0.163 |
| Note: Reference Interval Values = *W_l_* ± 0.1 | | | |
