# Supplementary material for: Simulating the Conversion of Rural Settlements to Town Land Based on Multi-Agent Systems and Cellular Automata
Source: PLoS One. 2013 Nov 11;8(11):e79300. doi: 10.1371/journal.pone.0079300 (PMC3823707; doi:10.1371/journal.pone.0079300)
Supplement: Table S6 — The decision weights of agents calculated by Delphi method. (DOC) [file pone.0079300.s007.doc]

| **Table S6. The decision weights of agents calculated by Delphi method.** | | | | | | | | | | | | | | | | | | |
| --- | --- | --- | --- | --- | --- | --- | --- | --- | --- | --- | --- | --- | --- | --- | --- | --- | --- | --- |
|  | | | | | | | | | | | | | | | | | | |
|  | **Yuyue** | | | | | | **Guanqiao** | | | | | | **Panjiawan** | | | | | |
|  | NL | | | R | | | NL | | | R | | | NL | | | R | | |
| N | G | I | F | G | I | F | G | I | F | G | I | F | G | I | F | G | I | F |
| 1 | 0.563 | 0.305 | 0.132 | 0.502 | 0.255 | 0.243 | 0.512 | 0.291 | 0.197 | 0.478 | 0.266 | 0.256 | 0.482 | 0.280 | 0.238 | 0.470 | 0.270 | 0.260 |
| 2 | 0.589 | 0.308 | 0.103 | 0.526 | 0.215 | 0.259 | 0.495 | 0.309 | 0.196 | 0.485 | 0.258 | 0.257 | 0.465 | 0.295 | 0.240 | 0.455 | 0.286 | 0.259 |
| 3 | 0.523 | 0.285 | 0.192 | 0.516 | 0.225 | 0.259 | 0.485 | 0.298 | 0.217 | 0.467 | 0.278 | 0.255 | 0.472 | 0.276 | 0.252 | 0.465 | 0.275 | 0.260 |
| 4 | 0.512 | 0.275 | 0.213 | 0.518 | 0.235 | 0.247 | 0.478 | 0.300 | 0.222 | 0.452 | 0.286 | 0.262 | 0.453 | 0.291 | 0.256 | 0.450 | 0.290 | 0.260 |
| 5 | 0.515 | 0.302 | 0.183 | 0.498 | 0.248 | 0.254 | 0.505 | 0.309 | 0.186 | 0.485 | 0.269 | 0.246 | 0.462 | 0.290 | 0.248 | 0.455 | 0.285 | 0.260 |
| 6 | 0.575 | 0.318 | 0.107 | 0.490 | 0.255 | 0.255 | 0.495 | 0.325 | 0.180 | 0.475 | 0.302 | 0.223 | 0.469 | 0.300 | 0.231 | 0.460 | 0.290 | 0.250 |
| 7 | 0.578 | 0.315 | 0.107 | 0.495 | 0.265 | 0.240 | 0.486 | 0.322 | 0.192 | 0.463 | 0.305 | 0.232 | 0.476 | 0.305 | 0.219 | 0.456 | 0.290 | 0.254 |
| 8 | 0.572 | 0.317 | 0.111 | 0.505 | 0.300 | 0.195 | 0.485 | 0.319 | 0.196 | 0.472 | 0.299 | 0.229 | 0.480 | 0.298 | 0.222 | 0.470 | 0.280 | 0.250 |
| 9 | 0.523 | 0.308 | 0.169 | 0.500 | 0.250 | 0.250 | 0.478 | 0.325 | 0.197 | 0.450 | 0.305 | 0.245 | 0.452 | 0.290 | 0.258 | 0.450 | 0.290 | 0.260 |
| 10 | 0.525 | 0.289 | 0.186 | 0.485 | 0.255 | 0.260 | 0.482 | 0.312 | 0.206 | 0.455 | 0.285 | 0.260 | 0.450 | 0.305 | 0.245 | 0.450 | 0.295 | 0.255 |
| 11 | 0.528 | 0.256 | 0.216 | 0.495 | 0.246 | 0.259 | 0.495 | 0.295 | 0.210 | 0.467 | 0.275 | 0.258 | 0.475 | 0.285 | 0.240 | 0.470 | 0.275 | 0.255 |
| 12 | 0.612 | 0.275 | 0.113 | 0.475 | 0.268 | 0.257 | 0.492 | 0.325 | 0.183 | 0.465 | 0.295 | 0.240 | 0.463 | 0.302 | 0.235 | 0.456 | 0.295 | 0.249 |
| 13 | 0.495 | 0.310 | 0.195 | 0.476 | 0.311 | 0.213 | 0.485 | 0.311 | 0.204 | 0.456 | 0.295 | 0.249 | 0.471 | 0.287 | 0.242 | 0.465 | 0.280 | 0.255 |
| 14 | 0.562 | 0.305 | 0.133 | 0.489 | 0.258 | 0.253 | 0.487 | 0.321 | 0.192 | 0.460 | 0.284 | 0.256 | 0.480 | 0.310 | 0.210 | 0.475 | 0.300 | 0.225 |
| 15 | 0.565 | 0.295 | 0.140 | 0.475 | 0.263 | 0.262 | 0.495 | 0.317 | 0.188 | 0.465 | 0.302 | 0.233 | 0.462 | 0.300 | 0.238 | 0.455 | 0.295 | 0.250 |
| 16 | 0.515 | 0.298 | 0.187 | 0.515 | 0.235 | 0.250 | 0.465 | 0.308 | 0.227 | 0.442 | 0.305 | 0.253 | 0.455 | 0.286 | 0.259 | 0.455 | 0.283 | 0.262 |
| 17 | 0.528 | 0.300 | 0.172 | 0.485 | 0.256 | 0.259 | 0.472 | 0.290 | 0.238 | 0.457 | 0.285 | 0.258 | 0.467 | 0.290 | 0.243 | 0.460 | 0.285 | 0.255 |
| 18 | 0.525 | 0.278 | 0.197 | 0.512 | 0.248 | 0.240 | 0.479 | 0.305 | 0.216 | 0.453 | 0.296 | 0.251 | 0.465 | 0.285 | 0.250 | 0.460 | 0.280 | 0.260 |
| 19 | 0.536 | 0.289 | 0.175 | 0.495 | 0.243 | 0.262 | 0.489 | 0.298 | 0.213 | 0.455 | 0.290 | 0.255 | 0.448 | 0.290 | 0.262 | 0.455 | 0.285 | 0.260 |
| 20 | 0.528 | 0.295 | 0.177 | 0.514 | 0.232 | 0.254 | 0.478 | 0.315 | 0.207 | 0.456 | 0.305 | 0.239 | 0.475 | 0.305 | 0.220 | 0.460 | 0.300 | 0.240 |
| 21 | 0.556 | 0.314 | 0.130 | 0.512 | 0.237 | 0.251 | 0.482 | 0.302 | 0.216 | 0.470 | 0.290 | 0.240 | 0.459 | 0.287 | 0.254 | 0.455 | 0.285 | 0.260 |
| **Table S6. Continued** | | | | | | | | | | | | | | | | | | |
|  | | | | | | | | | | | | | | | | | | |
|  | **Yuyue** | | | | | | **Guanqiao** | | | | | | **Panjiawan** | | | | | |
|  | NL | | | R | | | NL | | | R | | | NL | | | R | | |
| N | G | I | F | G | I | F | G | I | F | G | I | F | G | I | F | G | I | F |
| Mean | 0.544 | 0.297 | 0.159 | 0.499 | 0.252 | 0.249 | 0.487 | 0.309 | 0.204 | 0.463 | 0.289 | 0.247 | 0.466 | 0.293 | 0.241 | 0.459 | 0.286 | 0.254 |
| S.E. | 0.030 | 0.016 | 0.037 | 0.015 | 0.022 | 0.016 | 0.010 | 0.011 | 0.015 | 0.011 | 0.014 | 0.011 | 0.010 | 0.009 | 0.014 | 0.007 | 0.008 | 0.008 |
| Note: NL = Non-construction Land; R = Rural Settlements; N = Number; G = Government; I = Investors; F = Farmers; S.E. = Standard Errors. | | | | | | | | | | | | | | | | | | |
