# Supplementary material for: Simulating the Conversion of Rural Settlements to Town Land Based on Multi-Agent Systems and Cellular Automata
Source: PLoS One. 2013 Nov 11;8(11):e79300. doi: 10.1371/journal.pone.0079300 (PMC3823707; doi:10.1371/journal.pone.0079300)
Supplement: Table S7 — The variation of Kappa coefficient for 60 model realizations in the three towns. (DOC) [file pone.0079300.s008.doc]

| **Table S7. The variation of Kappa coefficient for 60 model realizations in the three towns.** | | | | |
| --- | --- | --- | --- | --- |
|  | | | | |
| **Number** | | **Yuyue** | **Guanqiao** | **Panjiawan** |
| 1 | | 0.7726 | 0.7038 | 0.7315 |
| 2 | | 0.7728 | 0.7042 | 0.7304 |
| 3 | | 0.7736 | 0.7029 | 0.7318 |
| 4 | | 0.7730 | 0.7033 | 0.7323 |
| 5 | | 0.7726 | 0.7026 | 0.7303 |
| 6 | | 0.7738 | 0.7033 | 0.7308 |
| 7 | | 0.7725 | 0.7038 | 0.7311 |
| 8 | | 0.7736 | 0.7028 | 0.7312 |
| 9 | | 0.7740 | 0.7035 | 0.7325 |
| 10 | | 0.7739 | 0.7026 | 0.7321 |
| 11 | | 0.7742 | 0.7023 | 0.7320 |
| 12 | | 0.7729 | 0.7036 | 0.7318 |
| 13 | | 0.7726 | 0.7041 | 0.7306 |
| 14 | | 0.7733 | 0.7037 | 0.7312 |
| 15 | | 0.7722 | 0.7038 | 0.7306 |
| 16 | | 0.7726 | 0.7029 | 0.7326 |
| 17 | | 0.7723 | 0.7031 | 0.7309 |
| 18 | | 0.7739 | 0.7029 | 0.7310 |
| 19 | | 0.7742 | 0.7033 | 0.7312 |
| 20 | | 0.7743 | 0.7032 | 0.7303 |
| 21 | | 0.7736 | 0.7026 | 0.7312 |
| 22 | | 0.7735 | 0.7023 | 0.7320 |
| 23 | | 0.7729 | 0.7033 | 0.7315 |
| 24 | | 0.7728 | 0.7037 | 0.7307 |
| 25 | | 0.7725 | 0.7040 | 0.7318 |
| 26 | | 0.7723 | 0.7036 | 0.7322 |
| 27 | | 0.7732 | 0.7028 | 0.7316 |
| 28 | | 0.7740 | 0.7030 | 0.7315 |
| 29 | | 0.7738 | 0.7031 | 0.7312 |
| 30 | | 0.7725 | 0.7029 | 0.7310 |
| 31 | | 0.7729 | 0.7034 | 0.7318 |
| 32 | | 0.7736 | 0.7036 | 0.7306 |
| 33 | | 0.7743 | 0.7043 | 0.7309 |
| 34 | | 0.7726 | 0.7032 | 0.7313 |
| 35 | | 0.7733 | 0.7036 | 0.7314 |
| 36 | | 0.7736 | 0.7029 | 0.7322 |
| 37 | | 0.7725 | 0.7030 | 0.7308 |
| 38 | | 0.7732 | 0.7038 | 0.7312 |
| 39 | | 0.7731 | 0.7025 | 0.7315 |
| 40 | | 0.7727 | 0.7034 | 0.7319 |
| **Table S7. Continued** | | | | |
|  | | | | |
| **Number** | | **Yuyue** | **Guanqiao** | **Panjiawan** |
| 41 | | 0.7735 | 0.7031 | 0.7310 |
| 42 | | 0.7728 | 0.7042 | 0.7309 |
| 43 | | 0.7734 | 0.7028 | 0.7313 |
| 44 | | 0.7738 | 0.7032 | 0.7305 |
| 45 | | 0.7736 | 0.7035 | 0.7318 |
| 46 | | 0.7724 | 0.7039 | 0.7304 |
| 47 | | 0.7731 | 0.7032 | 0.7321 |
| 48 | | 0.7729 | 0.7029 | 0.7309 |
| 49 | | 0.7735 | 0.7031 | 0.7310 |
| 50 | | 0.7725 | 0.7028 | 0.7315 |
| 51 | | 0.7728 | 0.7033 | 0.7317 |
| 52 | | 0.7726 | 0.7035 | 0.7309 |
| 53 | | 0.7728 | 0.7032 | 0.7305 |
| 54 | | 0.7735 | 0.7036 | 0.7319 |
| 55 | | 0.7732 | 0.7027 | 0.7322 |
| 56 | | 0.7725 | 0.7042 | 0.7315 |
| 57 | | 0.7730 | 0.7031 | 0.7306 |
| 58 | | 0.7726 | 0.7038 | 0.7316 |
| 59 | | 0.7733 | 0.7030 | 0.7311 |
| 60 | | 0.7729 | 0.7036 | 0.7313 |
| Mean | | 0.7731 | 0.7033 | 0.7313 |
| CIM | LB | 0.7730 | 0.7032 | 0.7312 |
|  | UB | 0.7733 | 0.7034 | 0.7315 |
| Minimum | | 0.7722 | 0.7023 | 0.7303 |
| Maximum | | 0.7743 | 0.7043 | 0.7326 |
| S.E. | | 0.0006 | 0.0005 | 0.0006 |
| CIM = 95% Confidence Interval for Mean; LB = Lower Bound; UB = Upper Bound; S.E. = Standard Errors. | | | | |
